# The Impact of Shielding Policy in Wales.

**DOI:** 10.23889/ijpds.v7i3.1791

**Published:** 2022-08-25

**Authors:** Tony Whiffen

**Affiliations:** 1 Welsh Government

## Objectives

Shielding was introduced as part of the UK government’s response to the SARS-CoV-2 pandemic to protect Clinically Extremely Vulnerable (CEV) people from infection and serious illness. Various research questions emerged in relation to non-clinical vulnerabilities of those shielding which could be addressed by utilising available health and administrative data.

## Approach

The Shielded Patient List (SPL) was linked with various datasets on the UK Secure Electronic Research Platform (UKSERP) including the Pupil Level Annual School Census (PLASC), National Survey and Ordnance Survey data for Wales. Some of these were anonymised datasets contained in the Secure Anonymised Information Linkage (SAIL) databank. Algorithms were applied to determine household composition and whether private outdoor space was available for the shielding group. Results were then extracted for Wales broken down by local authority.

## Results

Results from the various strands of research related to shielding will be presented covering provision of outdoor space, household characteristics and composition.

## Conclusion

These analyses demonstrate how population-level data resources can be leveraged quickly to answer newly-emerging policy questions as part of the response to the SARS-CoV-2 pandemic.

